# Cellular reprogramming and epigenetic rejuvenation

**DOI:** 10.1186/s13148-021-01158-7

**Published:** 2021-09-06

**Authors:** Daniel J. Simpson, Nelly N. Olova, Tamir Chandra

**Affiliations:** MRC Human Genetics Unit, MRC Institute of Genetics and Cancer, University of Edinburgh, Edinburgh, EH4 2XU UK

**Keywords:** Cellular reprogramming, Ageing, Rejuvenation, Epigenetic clocks, Transient reprogramming, Reprogramming-induced rejuvenation, Epigenetic age

## Abstract

Ageing is an inevitable condition that afflicts all humans. Recent achievements, such as the generation of induced pluripotent stem cells, have delivered preliminary evidence that slowing down and reversing the ageing process might be possible. However, these techniques usually involve complete dedifferentiation, i.e. somatic cell identity is lost as cells are converted to a pluripotent state. Separating the rejuvenative properties of reprogramming from dedifferentiation is a promising prospect, termed epigenetic rejuvenation. Reprogramming-induced rejuvenation strategies currently involve using Yamanaka factors (typically transiently expressed to prevent full dedifferentiation) and are promising candidates to safely reduce biological age. Here, we review the development and potential of reprogramming-induced rejuvenation as an anti-ageing strategy.

## Background

Ageing is a complex process that affects all humans, and with it comes an increased susceptibility to a range of diseases, tissue dysfunction and mortality [1, 2]. Many studies indicate that the ageing process may not be as inevitable as previously thought. Young blood has been shown to have rejuvenative properties through heterochronic parabiosis, whereby the circulatory systems of a young and old organism are connected [3–12]. The clearance of senescent cells in mice delays the onset of age-related pathologies and may extend life-span [13, 14]. Hence, drugs that selectively induce apoptosis in senescent cells (referred to as senolytics) have become a prominent topic of research in the molecular ageing field [15–17]. Manipulation of dietary intake is also one of the most well studied ageing interventions. Various diets (e.g. calorie restriction, intermittent fasting, ketogenic diet) manipulate nutrient sensing pathways, particularly those involving mTOR and insulin/insulin-like growth factor (IGF), to extend lifespan and reduce metabolic risk factors [17–26]. Various drugs, such as rapamycin, appear to mimic the effects of calorie restriction [27–32] and induce autophagy, a process the decline of which is associated with a number age-related diseases [17, 24, 33–35].

A recent addition to the anti-ageing strategies being developed, comes from cellular reprogramming approaches. Induced pluripotency studies provided evidence that age-related cellular phenotypes such as mitochondrial morphology, function and number, as well as nuclear envelope integrity, are not irreversible [17, 36–39]. However, developmental cellular reprogramming turns a cell to a pluripotent state, where it has the potential to generate any somatic cell type [40–42]. This process is not appropriate for an anti-ageing therapy in vivo because it requires not only the loss of the original cellular identity, but also the re-establishment of self-renewal capabilities. Therefore, induction of pluripotency or the direct injection of pluripotent cells in vivo, invariably lead to cancer in mice [43–46]. For a cellular reprogramming-based intervention to be considered rejuvenative (turning an old cell into a younger cell), we need to uncouple its effects from dedifferentiation (loss of somatic cell identity).

To separate rejuvenation from dedifferentiation, ageing trajectories and somatic cell identity must be analysed simultaneously. Ageing can be assessed by:

- epigenetic clocks; DNA methlyation-based age predictors created using penalised regression models, where a select group of CpGs that have a monotonically increasing relationship with age in a given training data are used to predict age [47]. Differences between the predicted epigenetic age (eAge) and chronological age (chAge) within individuals have been associated with diseases and environmental factors that appear to increase or decrease ageing at a physiological level [48–59].
  Hence, eAge has become a primary candidate metric for estimating biological age [60].
- gene transcription; analysis of key age-associated genes that increase or decrease in transcription with age and are associated with age-related outcomes [61–72]. A number of gene transcription-based clocks have been created in a similar manner to epigenetic clocks, but very few of them have been validated in other studies [68, 71–73].
- amelioration of physiological (e.g. declining organ function) and cellular (e.g. genomic integrity, mitochondrial health, nuclear envelope integrity, telomere length) ageing hallmarks [2, 74].

In this review, we will evaluate the current status of cellular reprogramming in the application of rejuvenation. We will also contextualise the efficacy of cellular reprogramming within the growing field of epigenetic age prediction.

## Cellular reprogramming demonstrates that age-related cellular changes are not irreversible

### Somatic cell nuclear transfer

In 1957, Conrad Waddington postulated that once a cell is fully differentiated, it cannot revert back to a pluripotent state [75]. The first evidence that cellular differentiation is malleable came shortly after with the development of somatic cell nuclear transfer (SCNT) [76, 77], where a somatic cell nucleus is transferred into an enucleated, unfertilized egg cell and divides to form an embryo that is genetically identical to the donor cell. Initial cloning experiments with SCNT were conducted with frogs [76, 77]. SCNT as a cloning process gained publicity when it was used to create the first ever cloned mammal, “Dolly” the sheep. An SCNT-derived artificial sheep zygote was implanted into a surrogate mother, resulting in the birth of a viable cloned sheep genetically identical to the initial donor [78]. One of the first questions raised was regarding the “age” of Dolly’s cells [79]. Did the biological age of Dolly’s cells match her chAge, or the chAge of her somatic donor? Indeed, the premature death of Dolly (aged 6.5 years) with normal life expectancy of 12 years for Dolly’s breed of sheep, combined with developing osteoarthritis [80, 81] raised concerns regarding Dolly’s biological age.

Telomere length was one of the main biomarkers available to measure age when Dolly was first created [82–85]. Analysis of Dolly’s cells revealed that the telomeres were actually shorter by ~20% compared to age-matched control sheep [86]. This observation initially suggested that SCNT does not reset biological age to zero [79]. However, analysis of telomeres of other SCNT-derived sheep (including sheep derived from the same cell line as Dolly) and other animals (e.g. mice) had normal telomere lengths for their respective age groups [80, 87–91]. Indeed, a recent study showed that SCNT of telomerase haplo-insufficient cells restores telomere length [92]. The exact reason Dolly had such anomalous health conditions remains a mystery, but as a proof-of-principle, SCNT showed that the reprogramming capabilities of the ovum may hold rejuvenative factors.

### Induced pluripotent stem cells

Groundbreaking work by Takahashi and Yamanaka in 2006 further proved that somatic cell identity is indeed rewritable. They showed that overexpression of four transcription factors (Oct3/4, Sox2, Klf4 and c-Myc, now referred to as the “Yamanaka factors” or “OSKM” factors) rearranges the epigenetic landscape and converts somatic cells to a pluripotent state [42, 93]. Since the creation of induced pluripotent stem cells (iPSCs) in vitro, it has become clear that cellular identity is dictated by epigenetic changes, rather than by loss or alterations of genomic DNA [94, 95]. The process of generating iPSCs has been optimised over the years, and has also been achieved via chemical induction, rather than forced gene expression, in mouse cells [96–98]. iPSCs﻿ offer the promise of directed, personalised regenerative therapy (i.e. iPSCs grown from patient cells, minimising incompatibility) for diseases that are currently incurable, such as neurodegenerative diseases of the central nervous system, heart infarction, diabetes mellitus, and also liver, lung, and kidney disease varieties [46]. However, ethical and safety considerations have to be met before iPSCs can be implemented for in vivo procedures [42, 46, 99], primarily regarding cancer risk.

After reprogramming, many signs of cellular ageing such as nuclear envelope integrity and mitochondrial morphology, function and number are improved [17, 36, 38, 39]. It has been proposed that as a cell converts to a pluripotent state, the aged epigenome is also reset to zero [94, 100]. Indeed, the Horvath epigenetic clock confirmed that ESCs and iPSCs have an eAge around zero [101] and it has recently been shown that in vivo, eAge reaches its “ground zero” state between E4.5–E10.5 in mice (a time-frame which encompasses the pluripotent state), after which organismal ageing begins [102, 103]. Telomeres of iPSCs are longer than in the parent differentiated cells, and are comparable in length to telomeres of control ESCs [104]. Telomere resetting even occurs when reprogramming somatic cells from both Hutchinson-Gilford progeria syndrome (HGPS) and supercentenarians [105].

## Reprogramming-induced epigenetic rejuvenation

**Fig. 1:**
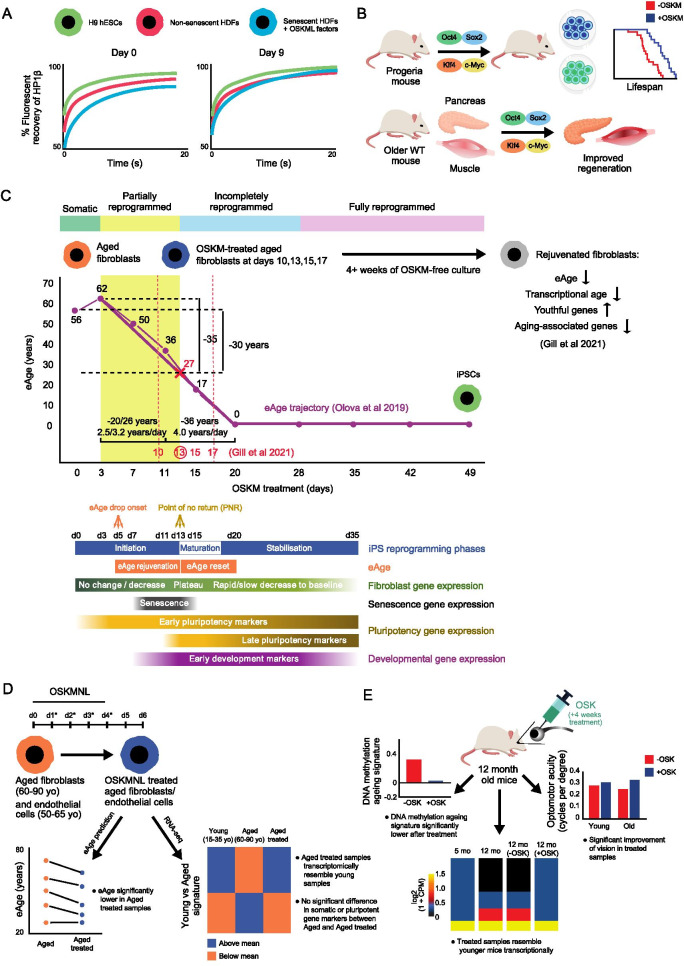
Cellular reprogramming experiments that induce epigenetic rejuvenation. **A** Fluorescent recovery of heterochromatin protein 1β (HP1β) in senescent cells was restored to the same level as non-senescent cells after nine days of OSKML treatment [119]. **B** OSKM treatment increased lifespan of progeria mice, improved the regenerative capacity of muscle and pancreas, as well as glucose tolerance [74]. **C** Overlaid summary of iPSC reprogramming time-course experiments by [118] and [73]. Upper panel: Horvath multi-tissue age predictor applied to OSKM-expressing adult fibroblasts [118]. The experimental setup of Gill et al. includes OSKM-free culturing at the time points in red for a minimum of four weeks. Day 13 was identified by Gill et al. as most suitable for OSKM withdrawal, where highest stable rejuvenation (eAge reduction) can be achieved: approximately 30 years, same as shown before OSKM withdrawal by Olova et al. Lower panel: The bars align with the time-course and summarise phases and patterns of gene expression as reported in Olova et al. “eAge drop onset” and “Point of no return” cannot be attributed to a precise day in the presented data as they occur between actual experimental time points. **D** OSKMNL-treatment of aged fibroblasts and endothelial cells results in a significant decrease in eAge (according to the Horvath clock), and a transcription profile resembling that of young fibroblasts/endothelial cells [125]. **E** 4 weeks of OSK treatment of 12 month old mice resulting in improved DNA methylation ageing signature, rejuvenation of age-related gene expression, and improved visual performance [127]

Reprogramming cells to pluripotency has shown that, in principle, age-related cellular phenotypes can be reversed, including in non-dividing, terminally differentiated cells [106–112]. However, this is based on dedifferentiation, turning cells into a stem-cell like state, as the underlying process. Dedifferentiation is also a process observed in oncogenesis [113–115]. To avoid the risk of cancer induction another strategy was proposed: epigenetic rejuvenation-where an old cell is made young again without a change of cell identity, i.e. dedifferentiation [94, 100, 116]. If, for example, the reversal of age during cellular reprogramming could be uncoupled from dedifferentiation, a viable rejuvenation strategy safe from cancer risk might exist. To achieve epigenetic rejuvenation via reprogramming factors, studies must first look at the intermediate states during dedifferentiation, where cells have started to epigenetically change (presumably de-age), but have not yet fully dedifferentiated [94, 100]. Partially reprogrammed cells are such examples, which are isolated between days 3 and 15 during classical human OSKM-induced dedifferentiation and have not yet lost their somatic identity [117, 118]. Therefore, partial reprogramming is a method of using OSKM factors (or alternative reprogramming factors, in the wider context) to revert aged cells to a younger state without completing the reprogramming cycle, thus retaining their cellular identity. A pilot attempt at conceptually testing epigenetic rejuvenation was made by Manukyan et al. where OSKM+LIN28 was expressed in human senescent fibroblasts and the mobility of heterochromatin protein 1β (HP1β) was restored to non-senescent levels, but not human embryonic stem cell (hESC) levels [119, 120], with the caveat that senescent/non-senescent are not the same as old/young fibroblasts (Fig. 1A). Furthermore, Ocampo et al. demonstrated that partial reprogramming, achieved by transient, periodic induction of OSKM (2 days on, then 5 days off, repeated several times), ameliorates signs of ageing without loss of cellular identity [74]. They conducted partial reprogramming first on progeroid (model for HGPS) mouse fibroblasts and alleviated age-associated hallmarks, such as DNA damage, nuclear envelope damage, dysregulation of histone modifications, stress and senescence associated factors, and mitochondrial-associated reactive oxygen species (ROS) production (cellular and epigenetic differences between aged and young cells and tissues are reviewed in detail in [121, 122]). Similar rejuvenation of dysregulated histone modifications was also observed when transient reprogramming was conducted on high-passage human fibroblasts (derived from iPSCs). Partial reprogramming was also applied in vivo to progeroid mice, which extended their lifespan in the absence of teratoma formation. When repeated in naturally aged mid-life mice, regenerative capacity of muscle and pancreas after injury was improved, as well as glucose tolerance (Fig. 1B; [74]).

Since Ocampo et al.’s transiently expressed OSKM in mice, partial reprogramming has become an exciting avenue for rejuvenation research. Unfortunately, eAge prediction for mouse was unavailable for Ocampo et al., hence the exact extent of rejuvenation by partial reprogramming in vivo could not be quantified. What also remained unclear was the nature of rejuvenation occurring. Was a subpopulation of cells dedifferentiating, or partially dedifferentiating and producing a rejuvenative effect to surrounding cells by being more stem-like? Alternatively, were the cells epigenetically rejuvenated, meaning that they became more youthful without loss of somatic cell identity?

To test these scenarios, two biomarkers were required to track (i) biological ageing and (ii) dedifferentiation state. We previously applied various eAge predictors to a well studied 49-day reprogramming time-course on human dermal fibroblasts (HDFs) that had both methylation data (to track eAge as a proxy for biological age) and gene expression data (to track cellular markers as a proxy for somatic identity) [118, 123]. This dataset was suitable to analyse since only cells expressing both the OSKM reporter (GFP) and TRA-1-60 (hESC marker) were flow sorted for analysis after day 3. Decline in eAge started after day 3 and steadily decreased until reaching eAge zero at day 20, well before the end of the reprogramming time-course (Fig. 1C). Meanwhile, fibroblast marker gene expression decreased but maintained comparatively stable levels until day 15, after which it dropped dramatically. The TRA-1-60(+) cell populations at days 7 and 11 were previously characterised as “partially reprogrammed” due to their high expression of pluripotency markers but also high reversion rates towards a somatic state [117]. This state, however, did not persist by day 15, when over 90 percent of cells could not spontaneously revert back to their somatic state after withdrawal of OSKM factors. Thus, the majority of day 15 cells were beyond the “point of no return” and into the more committed maturation phase of reprogramming, where the memory of their original cell identity is lost [117]. The “safe” from dedifferentiation partially reprogrammed state commenced before day 7 and ended between days 11 and 15 in the time-course, a window, which we provisionally positioned between days 3 and 13 [118] In this time-frame, the eAge of the partially reprogrammed cells already dropped dramatically by 30 to 35 years according to the Horvath clock (Fig. 1C). Thus, the partially reprogrammed state could provide a “safe window” where cells reach substantial epigenetic rejuvenation whilst retaining the ability to revert back to their original somatic identity.

Given the fixed number of time-points in Ohnuki et al.’s data, further investigation was required to firmly define the boundaries of a “safe” rejuvenation window. Another important question was whether the partially reprogrammed cells would retain their lower eAge and show a stable rejuvenated phenotype after reversion to somatic state, or would they quickly revert back to their original eAge? A recent preprint by Gill et al. addressed these questions using a similar in vitro reprogramming system with fibroblasts from middle aged donors and discontinuing OSKM expression after days 10, 13, 15 or 17 [73]. Importantly, the study confirmed a rejuvenated phenotype of the transiently reprogrammed cells, which is maintained upon reversion to somatic state at least four weeks after OSKM withdrawal. These cells retained lower eAge and showed lower transcriptional age, down-regulated age-associated gene expression and upregulated expression of genes characteristic for younger cells such as collagens (Fig. 1C). The authors defined day 13 as a “sweet spot” for rejuvenation, where eAge was reduced by approximately 30 years post OSKM withdrawal. This observation is consistent with the eAge reduction observed in the Ohnuki et al’s data for day 13 before OSKM withdrawal (through interpolation between time points), which also coincides with the partially reprogrammed “safe window” boundary defined in Olova et al. In addition, Gill et al. show that fibroblast-specific enhancers remained demethylated during the transient OSKM exposure, therefore acting as a carrier of epigenetic memory for fibroblast identity and facilitating the reversion to the somatic state. This supports our observation that during the partial reprogramming window, somatic genes maintain lower but stable expression, which does not drop to ESC and iPSC levels [118], pointing to a suppressed transcriptional program, which is "on hold" and not yet lost. An open question that neither of the two studies have firmly answered, remains the possibility that the de-ageing rate follows backwards the ticking rate of ageing throughout lifetime as measured by the Horvath clock, i.e. linear (slower) at first, followed by logarithmic (faster) before actual reset to zero (Fig. 1C, [101, 124]).

Other studies have confirmed and extended previous observations. Sarkar et al. transiently expressed OSKM+LIN28+NANOG (OSKMLN) in adult human dermal fibroblasts and endothelial cells for four days and analysed gene expression and methylation two days after interruption (Ocampo et al. by comparison used a doxycyclin-inducable system and forced expression 2–4 days in cell cultures; Fig. 1D; [125]). An important feature in this system is that OSKMLN is introduced non-integratively as a cocktail of mRNA molecules, meaning there is no random integration of OSMKLN in the genome, which minimises oncogenic risk. They compared the eAge and RNA expression of aged (60–90 year old) samples before and after treatment, with young (15–35 year old) samples. According to the Horvath clock, the OSKMNL treatment significantly reduced age in both the fibroblasts (mean age acceleration = − 1.84) and endothelial cells (mean age acceleration = − 4.94), although the effect was more pronounced in the latter. Another study developed a skin-specific eAge predictor and confirmed the rejuvenation of Sarkar et al’s OSKML treated fibroblasts [126]. In both tissues, RNA-seq analysis revealed that treated cells were transcriptionally comparable to younger cells than the original aged cells. This could be seen to a certain extent in a PCA analysis where in both tissues, treated cells tend to cluster closer to young cells than original aged cells. Rejuvenative effects were observed in analysis of other markers for heterochromatin, lamina, proteosomal activity, autophagosome formation and mitochondrial ROS. Additionally, expression of cell identity markers was maintained in the treated cells, meaning no loss of somatic identity occurred [125]. While adding significant evidence towards partial reprogramming as an epigenetic rejuvenation approach, no cell sorting based on pluripotency markers was conducted, which makes it difficult to place the transiently reprogrammed cells within the reprogramming trajectory of the other in vitro experiments.

Sarkar et al. went on to test the effect of partial reprogramming on stem cells by transplanting young, old, and transient OSKMNL-treated old mouse-derived skeletal muscle stem cells (MuSCs) into injured muscles of immunocompromised mice. They observed an improved regenerative ability in the OSKMNL-treated old MuSCs, comparable to that of the young MuSCs, and no teratomas or neoplastic lesions developed. Improved muscle function was also observed in the muscles grafted with OSKMNL-treated old MuSCs compared to untreated old MuSCs. Sarkar et al. repeated the experiment with old (60–80 years) human MuSCs, and found that they also had a higher proliferative capacity than the untreated cells, comparable to young human MuSCs [125].

Another cellular reprogramming approach has recently been tested in age- and injury-dependent impaired vision in mice [127]. Lu et al. showed that ectopic expression of *Oct4, Sox2* and *Klf4* (OSK) stimulated axon regeneration in an optic-nerve-crush-injury mouse model. The same strategy led to improved vision in a glaucoma mouse model. OSK treatment in healthy 12 month old mice improved visual acuity, and age-related gene expression more closely resembled that of young (4 or 5 month old) mice (Fig. 1E). An age-related DNA methylation signature of 1226 CpGs based on age, injury and OSK treatment was developed. A PCA of these CpGs was conducted using 14 control samples, which were used to create an “ageing signature”. According to this signature, the OSK-treated 12 month old mice ranked lower than the -OSK mice. Unfortunately, these results could not be replicated in aged (18 months) mice. Unlike Ocampo et al. who cyclically induced OSKM expression (continuous expression for 2 days out of repeated 7 day intervals), Lu et al. expressed OSK continuously. They excluded *c-Myc* from their treatment to avoid teratoma formation, since it is an oncogene [128]. In addition to its role in oncogenesis, reduced *c-Myc* expression also increases lifespan in mice [128]. After 10–18 months of continuous OSK expression in mice, no increase of tumour incidence was observed [127]. However, no direct measurement of cell identity or extent of dedifferentiation (e.g. somatic or pluripotency genes) was performed. It is therefore possible that cells are dedifferentiating, with the caveats mentioned above, rather than reversing their age.

During the revision of this manuscript, two preprints were released addressing cellular reprogramming and rejuvenation. A single two and a half weeks course of OSKM treatment in two months old heterozygous progeria mice increased lifespan and improved organ integrity, metabolism and motor skills [129]. These improvements were also maintained throughout the lifespan of the mice, pointing to a stably rejuvenated phenotype in time. Also, the first in vitro study utilising scRNA-seq showed that transient OSKM treatment restores a youthful transcriptomic ageing signature in both mouse adipogenic cells (adipocyte progenitors) and mesenchymal embryonic stem cells [130].

## Conclusion

Cellular reprogramming has demonstrated potential not only in regenerative medicine, but also in the ageing field through the amelioration of both physiological and cellular ageing hallmarks. While partial reprogramming might be used as a catch-all term to describe this type of rejuvenation, it does not reflect the fact that the described interrupted cellular reprogramming techniques are applied with the aim of (epigenetic) rejuvenation as opposed to inducing pluripotency (loss of cell identity). Reprogramming-induced rejuvenation (RIR) is a better term, capturing the nature of the utilised process and final aim of the interventions [131]. RIR has shown promise as a treatment to safely reverse ageing whilst retaining the ability to revert to or maintain original cell identity, both in vivo [74, 127, 129] and in vitro [73, 118, 125]. However, the precise nature of RIR still needs to be fully understood before it can be safely implemented as an anti-ageing treatment. For example, tracking any traces of pluripotency in partially reprogrammed cells (particularly in vivo) is a necessary precaution to minimise long-term cancer risk. Additionally, can rejuvenated partially reprogrammed cells be cultured long-term? According to Gill et al. the rejuvenated phenotype of their OSKM-treated cells lasts at least four weeks [73], but does this phenotype remain stable or eventually start to deteriorate at a rate faster than normal ageing?

Other important RIR safety concerns include how the reprogramming factors are introduced in vivo. Retroviruses are commonly used to integrate reprogramming factors into the genome [42, 93, 123]. However, this method bears risks, such as insertional mutagenesis, residual expression and re-activation of reprogramming factors, and retrotransposon activation, all of which could increase cancer risk in vivo [132, 133]. Non-integrative delivery methods, such as transient transfection, non-integrating viral vectors, and RNA transfection are safer alternatives [132]. For example, Sarkar et al. successfully used mRNA transfection to non-integratively conduct RIR [125]. Another safe alternative is chemical-based reprogramming, which involves direct conversion of a somatic cell to a pluripotent state by use of small molecules and growth factors [96–98]. It also avoids use of c-Myc, which is an oncogene [128]. It is conceivable that, in the future, chemical-based reprogramming could be adapted to achieve rejuvenation, however, this reprogramming approach currently only works for mice.

While RIR applied to skeletal muscle stem cells appears effective in improving regenerative capacity and muscle function in immunocompromised mice [125], further analysis is required regarding the somatic mosaicism of partially reprogrammed stem cells. Somatic variants at a stem or early progenitor cell level in turn can cause lineage bias, reduced stem cell function and increased risk of developing haematologic cancer (e.g. age-related clonal haematopoesis; [134–136]). This can lead to the development of pre-malignant cells, which have a higher propensity to transform to a malignant state [44, 45], the effect of which could be attenuated or exacerbated by RIR.

It also remains to be further explored whether and how RIR would work on post-mitotic terminally differentiated cells, such as neurons, cardiomyocytes or adipocytes, but also other non-dividing cells such as quiescent or senescent cells. Pilot work has been done in the latter two states [119, 125], demonstrating that a rejuvenated phenotype is achievable after restoration of cell division. These results may point to a scenario where proliferation is an essential requirement for rejuvenation. Indeed, induced pluripotency of postnatal neurons was only possible after forced cell proliferation via p53 expression [111]. Coincidentally, the natural rejuvenation event in the early mouse embryo spans over stages of very active cell proliferation (E4.5–E10.5) [103].

Overall, RIR is currently the best prospect to achieve epigenetic rejuvenation. Further studies are required to fully determine its limitations and efficacy.

## Data Availability

Not applicable.
